# Supplementary material for: The inner nuclear membrane protein Lem2 coordinates RNA degradation at the nuclear periphery
Source: Nat Struct Mol Biol. 2022 Sep 19;29(9):910–21. doi: 10.1038/s41594-022-00831-6 (PMC9507967; doi:10.1038/s41594-022-00831-6)
Supplement: Supplementary file 1 — Supplementary Tables 1–4 [file 41594_2022_831_MOESM1_ESM.pdf]

nature portfolio

https://doi.org/10.1038/s41594-022-00831-6

Supplementary information

The inner nuclear membrane protein Lem2 coordinates RNA degradation at the nuclear periphery

In the format provided by the authors and unedited

Supplementary Table 1 - *S. pombe* strains used in this study, related to experimental procedures

Strain	***Genotype***	**Source**	**Figure**
PSB0065	*P (h+) imr1L(NcoI)::ura4^{+} otr1R(SphI)::ade6^{+} leu1-32 ura4-DS/E ade6-M210*	(Braun et al., 2011)	1, ED1, 2b-h, ED2, ED3, ED4f, 5b-d, ED65a-c, 6, ED7c-f
PSB0906	*P (h+) leu1-32 ade6-210 ura4-DS/E imr1L(NcoI)::ura4^{+} otr1R(SphI)::ade6^{+} cen1::hphMX lem2::natMX*	(Barrales et al., 2016)	1, ED1, 2b-h, S2, 6, ED7c-e
PSB0642	*P (h-) leu1-32 ade6-210 ura4-D18 imr1L(NcoI)::ura4^{+} otr1R(SphI)::ade6^{+} mat1_m-cyhS smt0 rpl42::cyhR(sP56Q) cen1::hphMX man1::natMX*	(Barrales et al., 2016)	1f, ED1d
PSB0640	*P (h-) leu1-32 ade6-210 ura4-D18 imr1L(NcoI)::ura4^{+} otr1R(SphI)::ade6^{+} mat1_m-cyhS smt0 rpl42::cyhR(sP56Q) cen1::hphMX ima1::natMX*	(Barrales et al., 2016)	1f, ED1d
PSB0090	*P (h+) leu1-32 ade6-210 ura4-DS/E imr1L(NcoI)::ura4^{+} otr1R(SphI)::ade6^{+} clr4::natMX*	(Braun et al., 2011)	1g-h, ED1e, 2d
PSB1480	*P (h+) leu1-32 ade6-210 ura4-DS/E imr1L(NcoI)::ura4^{+} otr1R(SphI)::ade6^{+} clr3::kanMX*	(Barrales et al., 2016)	1g, ED1e
PSB1655	*M (h-) ura4-D18 leu1-32 ade6-M216 his7-366 swi6::natMX*	This study	1g, ED1e
PSB1729	*P (h+) leu1-32 ade6-210 ura4-DS/E imr1L(NcoI)::ura4^{+} otr1R(SphI)::ade6^{+} clr1::kanMX*	This study	1g, ED1e
PSB2486 (SPY418)	*P (h+) otr1R(SphI)::ura4^{+} ura4-DS/E leu1-32 ade6-M210 ago1::kanMX*	(Halic & Moazed, 2010)	1g, ED1e, 2d
PSB2487 (SPY1042)	*P (h+) leu1-32 ade6-M210 ura4DS/E otr1(SphI)::ura4^{+} oriA clr2Δ::TAP-kanMX*	(Motamedi et al., 2008)	1g, ED1e
PSB1813	*P (h+) imr1L(NcoI)::ura4^{+} otr1R(SphI)::ade6^{+} leu1-32 ura4-DS/E ade6-M210 rrp6::kanMX*	This study	ED1c, 2b-h, ED2a,d, ED3d-e
PSB2061	*P (h+) imr1L(NcoI)::ura4^{+} otr1R(SphI)::ade6^{+} leu1-32 ura4-DS/E ade6-M210 air1::kanMX*	This study	2b,d-e, ED2a,c, ED7c
PSB1888	*P (h+) imr1L(NcoI)::ura4^{+} otr1R(SphI)::ade6^{+} leu1-32 ura4-DS/E ade6-M210 erh1::kanMX*	This study	2b,d-e, ED2a,c, 6a
PSB1889	*P (h+) imr1L(NcoI)::ura4^{+} otr1R(SphI)::ade6^{+} leu1-32 ura4-DS/E ade6-M210 ccr4::kanMX*	This study	2b,d-e, ED2a,c
PSB1849	*P (h+) imr1L(NcoI)::ura4^{+} otr1R(SphI)::ade6^{+} leu1-32 ura4-DS/E ade6-M210 rrp6::kanMX lem2::natMX*	This study	2f, ED3e
PSB1784	*P (h+) imr1L(NcoI)::ura4^{+} otr1R(SphI)::ade6^{+} leu1-32 ura4-DS/E ade6-M210 red1::kanMX*	This study	ED1c, 2b,d-h, ED2a-b, ED3e
PSB1780	*P (h+) leu1-32 ade6-210 ura4-DS/E imr1L(NcoI)::ura4^{+} otr1R(SphI)::ade6^{+} cen1::hphMX lem2::natMX red1::kanMX*	This study	2f, ED3e
PSB1786	*P (h+) leu1-32 ade6-M210 ura4-DS/E imr1L(NcoI)::ura4^{+} otr1R(SphI)::ade6^{+} pab2::kanMX*	This study	2d,f, ED3e
PSB1781	*P (h+) leu1-32 ade6-210 ura4-DS/E imr1L(NcoI)::ura4^{+} otr1R(SphI)::ade6^{+} cen1::hphMX lem2::natMX pab2::kanMX*	This study	2f, ED3e
PSB1761	*P (h+) imr1L(NcoI)::ura4^{+} otr1R(SphI)::ade6^{+} leu1-32 ura4-DS/E ade6-M210 iss10::kanMX*	This study	2b,d-e, ED2a, 6b-c
PSB2651	*P (h+) imr1L(NcoI)::ura4^{+} otr1R(SphI)::ade6^{+} leu1-32 ura4-DS/E ade6-M210 red1^{+}-9xMyc::kanMX*	This study	ED3b, 5d, ED6a
PSB2653	*P (h+) leu1-32 ade6-210 ura4-DS/E imr1L(NcoI)::ura4^{+} otr1R(SphI)::ade6^{+} cen1::hphMX lem2::natMX red1^{+}-9xMyc::kanMX*	This study	ED3b, 5d, ED6a
PSB2694	*P (h+) leu1-32 ade6-210 ura4-DS/E imr1L(NcoI)::ura4^{+} otr1R(SphI)::ade6^{+} cen1::hphMX iss10::natMX red1^{+}-9xMyc::kanMX*	This study	ED3b

PSB0374	P (h+) leu1-32 ade6-216 ura4-D18	Bioneer haploid deletion library	ED1a-b, ED2a
PSB0593	P (h+) leu1-32 ade6-216 ura4-D18 man1::natMX	This study	ED1a, ED2a
PSB1487	P (h+) leu1-32 ade6-216 ura4-D18 clr4::kanMX	This study	ED1b, ED2a
PSB2410	M (h-) ura4-D18 leu1-32 ade6-M210 his7-366 red1^{+}-6xHA::kanMX6	This study	3a, ED7b
PSB2403	P (h+) leu1-32 ade6-210 ura4 lem2::natMX lem2^{+}-GFP::ura4^{+} red1^{+}-6xHA::kanMX6	This study	3a
PSB2703 (TM15)	M (h-) lys1^{+}::lem2p::lem2^{+}-GFP lem2::kanMX	This study	3d-e
PSB2704 (TM16)	M (h-) lys1^{+}::lem2p::lem2LC-GFP lem2::kanMX	This study	3d-e
PSB2705 (TM17)	M (h-) lys1^{+}::lem2p::lem2NL-GFP lem2::kanMX	This study	3d-e
PSB2706 (TM18)	M (h-) lys1^{+}::lem2p::lem2L-GFP Δlem2::kanMX	This study	3d-e
PSB2707 (TM19)	M (h-) lys1^{+}::lem2p::lem2(Δ200-307)-GFP lem2::kanMX	This study	3d-e
PSB2708 (TM20)	M (h-) lys1^{+}::lem2p-GFP Δlem2::kanMX	This study	3d-e
PSB2496	M (h-) ura4-D18 leu1-32 ade6-M210 his7-366 red1^{+}-6xHA::kanMX6 pREP81xLem2ΔN-GFP	This study	ED4a
PSB2498	M (h-) ura4-D18 leu1-32 ade6-M210 his7-366 red1^{+}-6xHA::kanMX6 pREP81xLem2ΔC-GFP	This study	ED4a
PSB2494	M (h-) ura4-D18 leu1-32 ade6-M210 his7-366 red1^{+}-6xHA::kanMX6 pREP81x-GFP	This study	ED4a
PSB2495	M (h-) ura4-D18 leu1-32 ade6-M210 his7-366 red1^{+}-6xHA::kanMX6 pREP81xLem2-GFP	This study	ED4a
PSB2453	P (h+) leu1-32 ade6-210 ura4 lem2^{+}-GFP::ura4^{+}::natMX	This study	ED4d-e, 5b, ED6a
PSB2914	P (h+) leu1-32 ade6-216 ura4-D18 kanMX::pGPD::Lem2-MSC soluble-GFP::ura4^{+} lem2::natMX	This study	ED4e
PSB2916	P (h+) leu1-32 ade6-216 ura4-D18 kanMX::pTEF::Lem2-MSC soluble-GFP::ura4^{+} lem2::natMX	This study	ED4d-f
PSB2831	P (h+) leu1-32 ade6-216 ura4-D18 lem2p::Lem2-MSC soluble-GFP::ura4^{+} lem2::natMX	This study	ED4d-f
PSB0591	P (h+) leu1-32 ade6-216 ura4-D18 lem2::natMX	(Barrales et al., 2016)	ED4f
PSB2840	P (h+) ade6-M216 ura4-D18 leu1-32 lem2^{+}-GFP::ura4^{+}::natMX	This study	ED4f
PSB2415 (JS71)	h^{90} ade6-M210 leu1 CO2::Padh1-4xU1A-Luc::natMX arg1::Padh41-U1Ap-YFP::hphMX	(Shichino, et al 2018)	4b, ED5b
PSB2416 (JS76)	h^{90} ade6-M210 leu1 CO2::Padh1-4xU1A-Luc-14xTTAAAC::natMX arg1::Padh41-U1Ap-YFP::hphMX	(Shichino et al., 2018)	4b, ED5b
PSB2446	h^{90} ade6-M210 leu1 CO2::Padh1-4xU1A-Luc-14xTTAAAC-natMX arg1::Padh41-U1Ap-YFP::hphMX cut11^{+}-mCherry::hphMX	This study	4b, ED5b-c
PSB2466	h^{90} ade6-M210 leu1 CO2::Padh1-4xU1A-Luc-14xTTAAAC::natMX arg1::Padh41-U1Ap-YFP::hphMX cut11^{+}-mCherry::hphMX lem2::kanMX	This study	4b, ED5c
PSB2918	h^{90} ade6-M210 leu1 CO2::Padh1-4xU1A-Luc-14xTTAAAC::natMX arg1::Padh41-U1Ap-YFP::hphMX cut11^{+}-mCherry::hphMX natMX::CFP-mmi1^{+}	This study	4c
PSB2919	h^{90} ade6-M210 leu1 CO2::Padh1-4xU1A-Luc-14xTTAAAC::natMX arg1::Padh41-U1Ap-YFP::hphMX cut11^{+}-mCherry::hphMX natMX::CFP-mmi1^{+} lem2::kanMX	This study	4c
H1N2330	M(h-) leu1-32 ura4-D18? sme2proxy[::ura4^{+}-kanMX6-lacOp] his7^{+}::lacI-GFP cut11^{+}-CFP::bsdMX	This study	ED5a
H1N2324	M(h-) leu1-32 ura4-D18? sme2proxy[::ura4^{+}-kanMX6-lacOp] his7^{+}::lacI-GFP cut11^{+}-CFP::bsdMX lem2::natMX	This study	ED5a

H1N2328	*M(h-) lys1-131 leu1-32 ura4-D18? sme2proxy[::ura4⁺-kanMX6-lacOp] his7⁺::lacI-GFP cut11⁺-CFP::bsdMX red1::natMX*	This study	ED5a
PSB2846	*h90 ade6-M210 leu1 CO2::Padh1-4xU1A-Luc-14xTTAAAC::natMX arg1::Padh41-U1Ap-YFP::hphMX cut11⁺-mCherry::hphMX iss10::kanMX*	This study	ED5b
PSB2844	*h90 ade6-M210 leu1 CO2::Padh1-4xU1A-Luc-14xTTAAAC::natMX arg1::Padh41-U1Ap-YFP-hphMX cut11⁺-mCherry::hphMX red1::kanMX*	This study	ED5b
PSB2044	*ura4-D18 leu1-32 ade6-M210 his7-366 cut11⁺-mCherry::hphMX siteF(Sme2)::kanMX-ura4⁺::lacOp his7⁺::LacI-GFP*	This study	ED5d
PSB2524	*P (h+) leu1-32 ade6-210 ura4 lem2⁺::natMX*	This study	5b, ED6a
PSB2662	*P (h+) imr1L(NcoI)::ura4⁺ otr1R(SphI)::ade6⁺ leu1-32 ura4-DS/E ade6-M210 natMX::GFP-mmi1⁺*	This study	ED3c, 5c, ED6a-c
PSB2664	*P (h+) imr1L(NcoI)::ura4⁺ otr1R(SphI)::ade6⁺ leu1-32 ura4-DS/E ade6-M210 natMX::GFP-mmi1⁺ lem2::kanMX*	This study	ED3c, 5c, ED6a-c
PSB2848	*P (h+) imr1L(NcoI)::ura4⁺ otr1R(SphI)::ade6⁺ leu1-32 ura4-DS/E ade6-M210 natMX::GFP-mmi1⁺ red1::kanMX*	This study	ED6b-c
PSB2030	*P (h+) leu1-32 ade6-210 ura4-DS/E imr1L(NcoI)::ura4⁺ otr1R(SphI)::ade6⁺ cen1::hphMX lem2::natMX erh1::kanMX*	This study	6a
PSB1765	*P (h+) leu1-32 ade6-210 ura4-DS/E imr1L(NcoI)::ura4+ otr1R(SphI)::ade6+ cen1::hphMX lem2::natMX iss10::kanMX*	This study	6b-c
PSB0044 (SPY139)	*h⁹⁰ leu1-32 ade6-M210 ura4DS/E mat3M::ura4⁺*	(Bühler, Haas, Gygi, & Moazed, 2007)	6d-e, ED7e
PSB0706	*h⁹⁰ leu1-32 ade6-M210 ura4DS/E mat3M::ura4⁺ lem2::natMX*	This study	6d-e
PSB2923	*P (h+) imr1L(NcoI)::ura4⁺ otr1R(SphI)::ade6⁺ leu1-32 ura4-DS/E ade6-M210 natMX::GFP-mmi1⁺ cut11⁺-mCherry::hphMX*	This study	ED7a
PSB2925	*P (h+) imr1L(NcoI)::ura4⁺ otr1R(SphI)::ade6⁺ leu1-32 ura4-DS/E ade6-M210 natMX::GFP-mmi1⁺ cut11⁺-mCherry::hphMX lem2::kanMX*	This study	ED7a
PSB1810	*P (h+) ura4-D18 leu1-32 ade6-M210 his7-366 cut11⁺-mCherry::hphMX red1⁺-GFP::kanMX*	This study	ED7a
PSB1837	*P (h+) ura4-D18 leu1-32 ade6-M210 his7-366 cut11⁺-mCherry::hphMX red1⁺-GFP::kanMX lem2::natMX*	This study	ED7a
PSB2042	*P (h+) ura4-D18 leu1-32 ade6-M210 his7-366 cut11⁺-mCherry::hphMX erh1⁺-GFP::kanMX*	This study	ED7a
PSB2094	*P (h+) ura4-D18 leu1-32 ade6-M210 his7-366 cut11⁺-mCherry::hphMX erh1⁺-GFP::kanMX lem2::natMX*	This study	ED7a
PSB2666	*P (h+) imr1L(NcoI)::ura4⁺ otr1R(SphI)::ade6⁺ leu1-32 ura4-DS/E? ade6-M210 his7-366 natMX::GFP-mmi1⁺ red1⁺-6xHA::kanMX6*	This study	ED7b
PSB2668	*P (h+) imr1L(NcoI)::ura4⁺ otr1R(SphI)::ade6⁺ leu1-32 ura4-DS/E? ade6-M210 his7-366 natMX::GFP-mmi1⁺ lem2::kanMX red1⁺-6xHA::kanMX6*	This study	ED7b
PSB2070	*P (h+) imr1L(NcoI)::ura4⁺ otr1R(SphI)::ade6⁺ leu1-32 ura4-DS/E ade6-M210 air1::kanMX lem2::natMX*	This study	ED7c
PSB2642	*P (h+) imr1L(NcoI)::ura4⁺ otr1R(SphI)::ade6⁺ leu1-32 ura4-DS/E ade6-M210 sme2::natMX*	This study	ED7d
PSB2675	*P (h+) imr1L(NcoI)::ura4⁺ otr1R(SphI)::ade6⁺ leu1-32 ura4-DS/E ade6-M210 sme2::natMX lem2::kanMX*	This study	ED7d

Supplementary table 4 - *S. cerevisiae* strains used in this study, related to experimental procedures

Strain	Genotype	Source	Figure
SGY137	Y2H gold (*MATa, trp1-901, leu2-3, 112, ura3-52, his3-200, gal4Δ, gal80Δ, LYS2::GAL1*$_{UAS}$*–GAL1*$_{TATA}$*::HIS3, GAL2*$_{UAS}$*–GAL2*$_{TATA}$*::Ade2 URA3::MEL1*$_{UAS}$*–MEL1*$_{TATA}$*::AUR1-C MEL1*)	Clontech	3b, ED4b

Supplementary table 5 - Plasmids used in this study, related to experimental procedures

Bacterial host strain	Plasmid	Insert	Marker	Source	Figure
ESB617	pJK210-lem2p-Lem2-GFP-lem2t	Lem2-GFP	ampR *ura4*$^{+}$	This study	3a, ED4d-f
ESB472	pGADT7	GAL4 AD	ampR *leu2*$^{+}$	Clontech	3b
ESB469	pGBKT7	GAL4-DBD	kanR *trp1*$^{+}$	Clontech	3b
ESB532 (368)	pGADT7	GAL4 AD spRed1-1-712	ampR *leu2*$^{+}$	(Dobrev et al., 2021)	3b, ED4b
ESB644	pGADT7	GAL4 AD hsRed1-492-1308	ampR *leu2*$^{+}$	This study	3b
ESB476	pGBKT7	GAL4-DBD spLem2-MSC-568-688	kanR *trp1*$^{+}$	This study	3b
ESB629	pGBKT7	GAL4-DBD hsLem2-MSC-395-503	kanR *trp1*$^{+}$	This study	3b
ESB543	pGADT7	GAL4 AD spRrp6-1-777	kanR *trp1*$^{+}$	This study	3b, ED4b
ESB379	pREP 81xLem2ΔN-GFP	Lem2ΔN-GFP	ampR *leu2*$^{+}$	(Barrales et al., 2016)	ED4a
ESB380	pREP 81xLem2ΔC-GFP	Lem2ΔC-GFP	ampR *leu2*$^{+}$	(Barrales et al., 2016)	ED4a
ESB382	pREP 81xLem2-GFP	Lem2-GFP	ampR *leu2*$^{+}$	(Barrales et al., 2016)	ED4a
ESB621	pREP 81xGFP	GFP	ampR *leu2*$^{+}$	(Forsburg, 1993)	ED4a
ESB533 (436)	pGADT7	GAL4 AD spIss10-1-551	kanR *trp1*$^{+}$	(Dobrev et al., in press)	ED4b
ESB545 (438)	pGADT7	GAL4 AD spPab2-1-166	kanR *trp1*$^{+}$	(Dobrev et al., in press)	ED4b
ESB546 (369)	pGBKT7	GAL4-DBD spMtl1-1-1030	kanR *trp1*$^{+}$	(Dobrev et al., in press)	ED4b
ESB614	pJK210-lem2p-NLS-MSC-GFP-lem2t	NLS-MSC-GFP	ampR *ura4*$^{+}$	This study	ED4d-f

Supplementary table 6 - Primer sets used for RT-qPCR, ChIP-qPCR, RIP-qPCR, and northern probes related to experimental procedures

oligo name	FOR oligo	REV oligo	locus	reference
Sg1030/1031 (P638/639)	AACCCTCAGCTTTGGGTCTT	TTTGCATACGATCGGCAATA	*act1+*	(Braun et al., 2011)
Sg2736/2737	TGGCCTTCTTAGCCTTTTCA	CTGAGGAAGTTTGGGCTGTC	*tef3+*	(Georgescu et al. 2020)
Sg2670/2671	AGGCATCTGATCCCAATGAG	ATTTTGGATGCCTTGGATGA	*ade2+*	(Georgescu et al., 2020)
Sg1020/1021 (P059/060)	TGCTCTGACTTGGCTTGTCTT	CCCTAACTTGGAAAGGCACA	*cen-dg*	(Braun et al., 2011)
Sg1886/1887 (P_F'tlh1- mb274/276)	ATGGTCGTCGCTTCAGAAATTGC	CTCCTTGGAAGAATTGCAAGCCTC	*tlh1+*	(Bühler et al., 2007)
Sg3493/3494	TGGTCAATCTTCTGCCGTCT	ACGAACAAACCACAACACAAAG	*sme2+*	This study
Sg2475/2476	CATGGAATGGTTTGAGCGTCA	CTGAACTACCGACCCACAGA	*sno20+*	This study
Sg2230/2231	CCACTACGTCCATCATCCCG	AGCGTAGGACTTGAAGGTGC	*mei4+*	This study
Sg2324/2325	TGCTTTACCAAATCGCACCA	CGCTATGTACCGATCCAGCT	*mei3+*	This study
Sg3463/3464	GGCCAGCTGCTTTTCTACTG	ACAGCTAAAGACCGCAAGGA	*ssm4+*	This study
Sg3678/3679 (ssm4-qPCR-fwd/rev)	CAGTTACTAATATCTTCTCAACCTG	GCACTGTTTAACTCGTCTATTAC	*ssm4+*	(Xie et al., 2019)
Sg2944/2945	TAATGAGTTGCCCCGGGTAT	CCGAATGGCAAGATGGTAAT	*SPAC212.08c*	(Georgescu et al., 2020)
Sg4099/4100	CTGCACCGATGCTGTTTCATC	TTTGAGGCGTCAGGTTTGTTG	*dic1+*	This study
Sg4085/4086	ACCAAGACAAGACGATTCACGA	TGTGGTATGGCAGAGGGTTTG	*meu1+*	This study
Sg3465/3466	ATCCAAAAATAGCGCCAATG	ATTTCCTTGTCGACGGTGTC	*mcp5+*	This study
Sg3684/3685 (rec8-2-qPCR-fwd/rev)	TACCGTTACCCGTTCCATTG	CCATTGGGACAAAGTTCGAG	*rec8+*	(Xie et al., 2019)
Sg3802/3803	TGTCGGAAGGTAATCGCACAA	CCGTTTCAGGCTCCCAGTATT	*mug8+*	This study
Sg4093/4094	ATGAGAAAGCAAGCAGGTGGT	TCCCGAATAATCACGACGGAC	*mug9+*	This study
Sg4109/4110	CTTCTTCGTGCAACAGTCAGC	GTTTTGACCGCCATCCGAAAA	*mug10+*	This study
Sg4091/4092	TGTGTCAACCTCGAACACAGT	TTCAAACGCCTCGCACAATTT	*tht2+*	This study
Sg3461/3462	ATTGGCATCATTTTCGGTTC	CGGAAAAGATTGGCACTAGC	*mcp7+*	This study
Sg4079/4080	CAGAGACAACCATCCGACCAA	GTGCAGGTAGGAGAAGCAGAT	*mug45+*	This study
Sg4097/4098	TATTGGCACTGTATGCCTCCG	CTGTAACGTCACGACCTCCAA	*arp1+*	This study
Sg4077/4078	CGTGTAGGTGAAGTGCAGTCT	AAGCTTTGGAGCCCAACTGTA	*mug4+*	This study
Sg4107/4108	GAAGCCCGTTTGGCTCAAAAA	AACACGTTAGCAGCCCTTGTA	*rec25+*	This study
Sg4089/4090	ACCATGTCCTGGGTTTGGTAG	ATACATTGACGAACGCCCCAT	*meu32+*	This study
Sg4081/4082	GGTCCGATGCTAATGGTTTGC	CGGCGGTGTTTTGAAATCAGT	*rep1+*	This study
Sg4105/4106	GGAGCCAGACTCAAGTAGCAG	TCCTCGAGCTTGACAGTTTCC	*rec10+*	This study
Sg4075/4076	TTCCCAACTTCCCTTCGTTGA	TGAACCCTGCCAACTGCTAAT	*mug1+*	This study
Sg4087/4088	AGGCCATTGTTAAACATGCGA	TGCCATGGCTTCAGTCAAGAT	*meu43+*	This study
Sg4103/4104	GGCTTGCATACCTCAGTCGAT	TATTCCAGCTTGTTGGGTCCG	*dil1+*	This study
Sg4083/4084	GGACGTTCACTCACCGGATAA	AAATCGGCGAAGGTCTTCTGT	*mcp6+*	This study
Sg1573/Sg1574	CGAGACCCCCTAATGCTTTT	CCAGGGTACATTTTCTGATGTTG	*mat3M*	(Barrales et al., 2016)
Sg3469/3470	GTCCGCTTGTAGCGCTGTA	TTCCAGATTCAATCAAGCACA	*Tf2-5*	This study

Sg2477/2478	CGGTATAGATTATGTCGGGGAGA	CGTAGCATTCGGTCATCACG	*snR42*$^{+}$	This study
Sg3970/3971	GGTTGCAAAACGCTTCCATCT	TATCATCCCCCTCGGGTGTAA	*luc*	This study
Sg4123/4124	CGTTTTGCAGCAGACTCGAAA	AGATATCGCGCTGATTCCAGG	*lem2*$^{+}$	This study
P249/250	TGGATCAGATCCGTGGAATC	AACGCTCGATTAGAAGGCAT	*mei4*$^{+}$	(Simonetti et al., 2017)
P325/326	ACACAGTTTACGGGATTCTA	GATTGTGATGAAAACTGGGT	*ssm4*$^{+}$	(Simonetti et al., 2017)
P1836/1838	ATCGTGCGGTTATATACCGAC	CACTAAGATGTCCAATATGGCTC	*sno20*$^{+}$	This study
P1839/1840	TTCATGTTATGTGTTGGTTGTATTTG	CAGGTTTTTAAAACCCACGAATAC	*sno20*$^{+}$ *3'*-extended	This study
P1841/1843	CTGATGTCAAAAAGTCTGCTCTC	GTCTCCCCGACATAATCTATACC	*snR42*$^{+}$	This study
P1844 /1845	GTTATTGTTGTAGTTAAAATACGTTG	GTACATGAGACATATGATGCTTAC	*snR42*$^{+}$ *3'*-extended	This study
P1836 /1837	ATCGTGCGGTTATATACCGAC	TAATACGACTCACTATAGGG GCTATCTGAACTACCGACCCA	*sno20*$^{+}$ (northern)	This study
P1841 /1842	CTGATGTCAAAAAGTCTGCTCTC	TAATACGACTCACTATAGGG TGTACGTAGCATTCGGTCATC	*snR42*$^{+}$ (northern)	This study

References:

Barrales, R. R., Forn, M., Georgescu, P. R., Sarkadi, Z., & Braun, S. (2016). Control of heterochromatin localization and silencing by the nuclear membrane protein Lem2. *Genes & Development*, *30*(2), 133–148. http://doi.org/10.1101/gad.271288.115

Braun, S., Garcia, J. F., Rowley, M., Rougemaille, M., Shankar, S., & Madhani, H. D. (2011). The Cul4-Ddb1(Cdt)^2 ubiquitin ligase inhibits invasion of a boundary-associated antisilencing factor into heterochromatin. *Cell*, *144*(1), 41–54. http://doi.org/10.1016/j.cell.2010.11.051

Bühler, M., Haas, W., Gygi, S. P., & Moazed, D. (2007). RNAi-dependent and -independent RNA turnover mechanisms contribute to heterochromatic gene silencing. *Cell*, *129*(4), 707–721. http://doi.org/10.1016/j.cell.2007.03.038

Ding, D.-Q., Matsuda, A., Okamasa, K., Nagahama, Y., Haraguchi, T., & Hiraoka, Y. (2016). Meiotic cohesin-based chromosome structure is essential for homologous chromosome pairing in Schizosaccharomyces pombe. *Chromosoma*, *125*(2), 205–214. http://doi.org/10.1007/s00412-015-0551-8

Dobrev, N., Ahmed Y.L., Sivadas, A, Soni, K., Fischer, T., Sinning, I. (2021). The zinc-finger protein Red1 orchestrates MTREC submodules and binds the Mtl1 helicase arch domain. *Nat Commun* 12, 3456.

Forsburg, S. L. (1993). Comparison of Schizosaccharomyces pombe expression systems. *Nucleic Acids Research*, *21*(12), 2955–2956. http://doi.org/10.1093/nar/21.12.2955

Georgescu, P. R., Capella, M., Fischer-Burkart, S., & Braun, S. (2020). The euchromatic histone mark H3K36me3 preserves heterochromatin through sequestration of an acetyltransferase complex in fission yeast. *Microbial Cell (Graz, Austria)*, *7*(3), 80–92. http://doi.org/10.15698/mic2020.03.711

Halic, M., & Moazed, D. (2010). Dicer-independent primal RNAs trigger RNAi and heterochromatin formation. *Cell*, *140*(4), 504–516. http://doi.org/10.1016/j.cell.2010.01.019

Motamedi, M. R., Hong, E.-J. E., Li, X., Gerber, S., Denison, C., Gygi, S., & Moazed, D. (2008). HP1 proteins form distinct complexes and mediate heterochromatic gene silencing by nonoverlapping mechanisms. *Molecular Cell*, *32*(6), 778–790. http://doi.org/10.1016/j.molcel.2008.10.026

Shichino, Y., Otsubo, Y., Kimori, Y., Yamamoto, M., & Yamashita, A. (2018). YTH-RNA-binding protein prevents deleterious expression of meiotic proteins by tethering their mRNAs to nuclear foci. *eLife*, *7*, 999. http://doi.org/10.7554/eLife.32155

Simonetti, F., Candelli, T., Leon, S., Libri, D., & Rougemaille, M. (2017). Ubiquitination-dependent control of sexual differentiation in fission yeast. *eLife, 6*, e28046. http://doi.org/10.7554/eLife.28046

Xie, G., Vo, T. V., Thillainadesan, G., Holla, S., Zhang, B., Jiang, Y., et al. (2019). A conserved dimer interface connects ERH and YTH family proteins to promote gene silencing. *Nature Communications*, *10*(1), 251. http://doi.org/10.1038/s41467-018-08273-9
