# Supplementary material for: The inner nuclear membrane protein Lem2 coordinates RNA degradation at the nuclear periphery
Source: Nat Struct Mol Biol. 2022 Sep 19;29(9):910–21. doi: 10.1038/s41594-022-00831-6 (PMC9507967; doi:10.1038/s41594-022-00831-6)
Supplement: Supplementary file 2 — Reporting Summary [file 41594_2022_831_MOESM2_ESM.pdf]

nature portfolio

Corresponding author(s): Dr. Sigurd Braun

Last updated by author(s): Jun 12, 2022

Reporting Summary

Nature Portfolio wishes to improve the reproducibility of the work that we publish. This form provides structure for consistency and transparency in reporting. For further information on Nature Portfolio policies, see our Editorial Policies and the Editorial Policy Checklist.

Statistics

For all statistical analyses, confirm that the following items are present in the figure legend, table legend, main text, or Methods section.

n/a	Confirmed	
☐	☒	The exact sample size (*n*) for each experimental group/condition, given as a discrete number and unit of measurement
☐	☒	A statement on whether measurements were taken from distinct samples or whether the same sample was measured repeatedly
☐	☒	The statistical test(s) used AND whether they are one- or two-sided<br>*Only common tests should be described solely by name; describe more complex techniques in the Methods section.*
☒	☐	A description of all covariates tested
☐	☒	A description of any assumptions or corrections, such as tests of normality and adjustment for multiple comparisons
☐	☒	A full description of the statistical parameters including central tendency (e.g. means) or other basic estimates (e.g. regression coefficient) AND variation (e.g. standard deviation) or associated estimates of uncertainty (e.g. confidence intervals)
☐	☒	For null hypothesis testing, the test statistic (e.g. *F*, *t*, *r*) with confidence intervals, effect sizes, degrees of freedom and *P* value noted<br>*Give P values as exact values whenever suitable.*
☒	☐	For Bayesian analysis, information on the choice of priors and Markov chain Monte Carlo settings
☒	☐	For hierarchical and complex designs, identification of the appropriate level for tests and full reporting of outcomes
☐	☒	Estimates of effect sizes (e.g. Cohen's *d*, Pearson's *r*), indicating how they were calculated

Our web collection on statistics for biologists contains articles on many of the points above.

Software and code

Policy information about availability of computer code

Data collection	Live-cell microscopy images were acquired with either a Zeiss AxioObserver Z1 confocal spinning disc microscope with an EMM-CCD camera, or an inverted Leica SP8X WLL microscope, equipped with 405 nm laser, WLL2 laser (470 - 670 nm) and acusto-optical beam splitter or a DeltaVision Elite system equipped with pco.edge 4.2 sCMOS camera.<br>For smFISH, images were acquired with a DeltaVision Elite system (GE Healthcare Inc.) equipped with pco.edge 4.2 sCMOS camera (PCO). Chromatic shifts were corrected using Chromagnon software (v0.87) using a bleed-through fluorescence image as a reference. The images were denoised by the ND-safir program 73, deconvolved using the built-in SoftWoRx software (v7.0.0), and then projected by maximum intensity projection<br>RNAseq libraries were generated with NEBNext Ultra Directional RNA Library (Illumina) and sequenced on HiSeq1500 or NextSeq 2000.<br>Individual RT, RIP and ChIP-samples were analyzed by quantitative real-time PCR using QuantStudioTM 3 or QuantStudioTM 5 (Thermo Fisher) real-time PCR systems and processed using Thermo Fisher ConnectTM. For quantitative snoRNA analysis, a LIghtCycler LC480 (Roche) was used.
Data analysis	FiJi/ImageJ 2.1.0/1.53c (regularly updated) were used for confocal image analysis.<br>Graphpad Prism 8 was used for data plotting of qPCR data.<br>RNAseq data were analyzed using the following packages: STAR v2.7.3a; RSEM v1.3.3 ; DESeq2 v1.22.2; Tximport v1.10.1; sva v3.30.1; Bedtools v2.29.1.<br>The Bähler Lab AnGeLi tool (www.bahlerlab.info/AnGeLi) was used for the analysis of gene lists from RNAseq data.

For manuscripts utilizing custom algorithms or software that are central to the research but not yet described in published literature, software must be made available to editors and reviewers. We strongly encourage code deposition in a community repository (e.g. GitHub). See the Nature Portfolio guidelines for submitting code & software for further information.

Data

Policy information about availability of data

All manuscripts must include a data availability statement. This statement should provide the following information, where applicable:

- Accession codes, unique identifiers, or web links for publicly available datasets
- A description of any restrictions on data availability
- For clinical datasets or third party data, please ensure that the statement adheres to our policy

All sequencing data that support the findings of this study have been deposited in the National Center for Biotechnology Information Gene Expression Omnibus (GEO) and are accessible through the GEO Series accession number GSE174347. Full code for all NGS-related workflow is available at: https://github.com/Tsvanemden/Martin_Caballero_et_al_2021.

Field-specific reporting

Please select the one below that is the best fit for your research. If you are not sure, read the appropriate sections before making your selection.

☒ Life sciences ☐ Behavioural & social sciences ☐ Ecological, evolutionary & environmental sciences

For a reference copy of the document with all sections, see nature.com/documents/nr-reporting-summary-flat.pdf

Life sciences study design

All studies must disclose on these points even when the disclosure is negative.

Sample size	No statistical methods were performed to predetermine the sample size, but the number of biological replicates for each experiment was based on similar studies (Barrales et al., 2016, PMID: 26744419; Ding et al., 2019, PMID: 31811152; Egan et al., 2014, PMID: 24713849; Shichino et al., 2018, PMID: 29424342; Sugiyama et al., 2016, PMID: 26942678; Thillainadesan et al., 2020, PMID: 32415063; Wei et al., 2021, PMID: 33574613).
Data exclusions	No data were excluded from the analysis unless they were classified as outliers based on the Grubbs' test using the outliers calculator provided by graphpad (https://www.graphpad.com/quickcalcs/Grubbs1.cfm) with a significant level of alpha = 0.05
Replication	For all experiments, at least two independent biological replicates were performed and each replicate was reliably reproduced.
Randomization	For all experiments (ChIP, coIP, RIP, RNAseq, RT-qPCR, WB, Y2H), yeast cultures were grown under the same conditions and collected randomly without any bias. Microscopy data were generated from randomly selected cells (DIC channel) across several fields before analysis in the respective fluorescence channel.
Blinding	Blinding strategies are not relevant in the context of the experiments presented.

Reporting for specific materials, systems and methods

We require information from authors about some types of materials, experimental systems and methods used in many studies. Here, indicate whether each material, system or method listed is relevant to your study. If you are not sure if a list item applies to your research, read the appropriate section before selecting a response.

Materials & experimental systems

n/a	Involved in the study	
☐	☒	Antibodies
☒	☐	Eukaryotic cell lines
☒	☐	Palaeontology and archaeology
☒	☐	Animals and other organisms
☒	☐	Human research participants
☒	☐	Clinical data
☒	☐	Dual use research of concern

Methods

n/a	Involved in the study	
☒	☐	ChIP-seq
☒	☐	Flow cytometry
☒	☐	MRI-based neuroimaging

Antibodies

Antibodies used	- anti-HA (3F10), Rat monoclonal, Roche Cat# 11867423001, diluted 1:1,000 for western blot.<br>- anti-GFP (B-2), Mouse monoclonal, Santa Cruz Biotechnology Cat# sc-9996, diluted 1:1,000 for western blot.<br>- anti-Myc, Rabbit polyclonal, Abcam Cat# ab9106, diluted 1:2,000 for western blot.<br>- anti-H3 (1B1-B2), Mouse monoclonal, Merck Cat# MABE923, diluted 1:5,000 for western blot.<br>- anti-Rat IgG/HRP conjugate, Goat polyclonal, Merck Millipore Cat# AP136P, diluted 1:3,000 for western blot.

- anti-Mouse IgG/HRP conjugate, Goat polyclonal, BioRad Cat# 170-6516, diluted 1:3,000 for western blot.
- anti-Rabbit IgG/HRP conjugate, Goat polyclonal, BioRad Cat# 170-6515, diluted 1:3,000 for western blot.
- GFP-Trap Agarose, Chromotek Cat# gta-100, for immunoprecipitation.
- Myc-Trap Agarose, Chromotek Cat# yta-100, for immunoprecipitation.
- anti-H3K9me2, Mouse monoclonal, Abcam Cat# ab1220, 2 µg for immunoprecipitation.
- anti-Pol II-S5P CDT 3E8, Mouse monoclonal (generated by the lab of Dirk Eick), for immunoprecipitation.

Validation

All antibodies are commercially available and were validated by the manufactures, except for the anti-Pol II-S5P antibody, which was validated by Dirk Eick Lab.
