# Supplementary figures and images for: The inner nuclear membrane protein Lem2 coordinates RNA degradation at the nuclear periphery
Source: Nat Struct Mol Biol. 2022 Sep 19;29(9):910–21. doi: 10.1038/s41594-022-00831-6 (PMC9507967; doi:10.1038/s41594-022-00831-6)

## Uncropped gel images

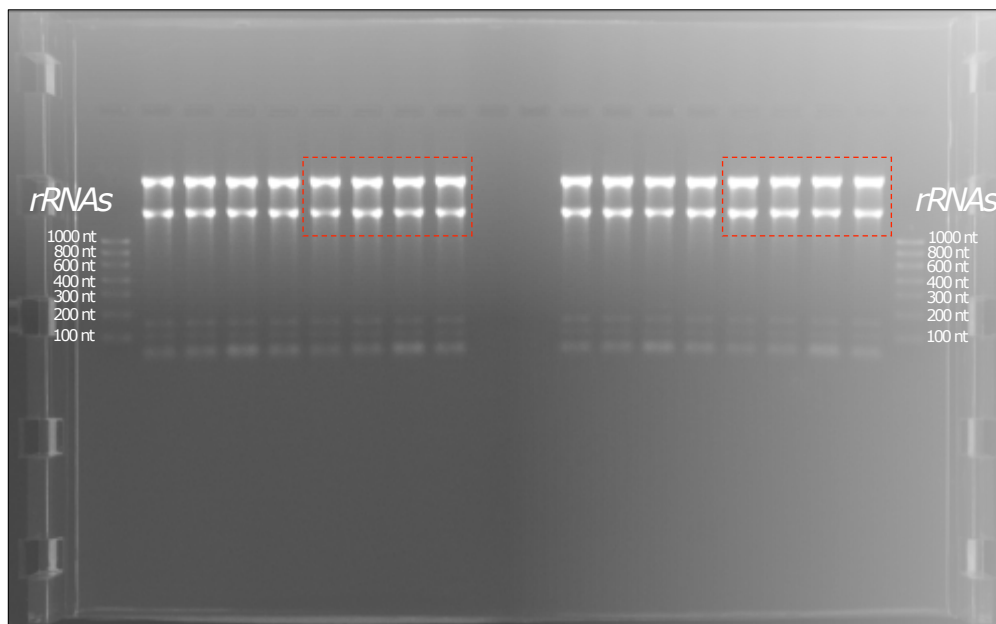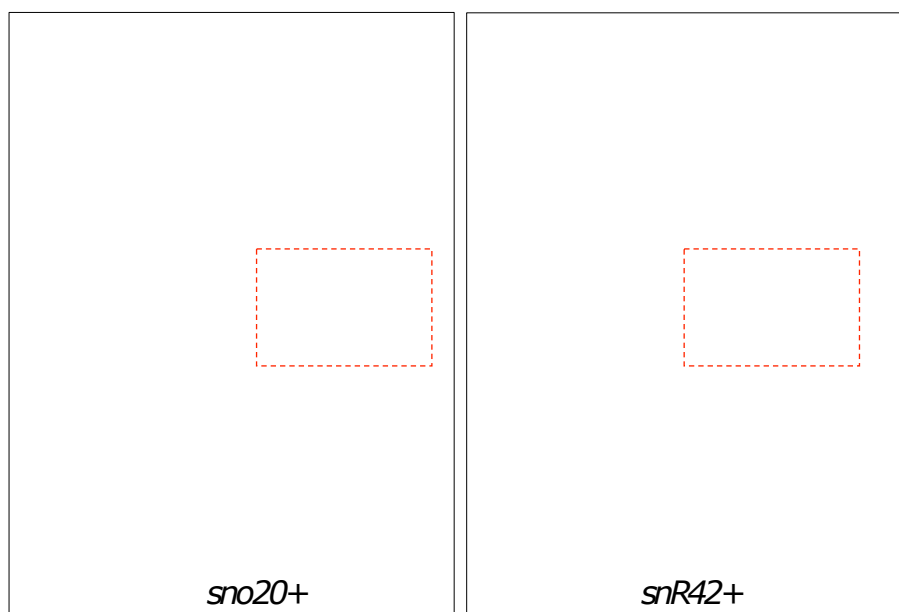

**Fig. 2h**

Supplement: Source Data Fig. 2 — Unprocessed gels [file 41594_2022_831_MOESM8_ESM.pdf]

# HA (Red1) input

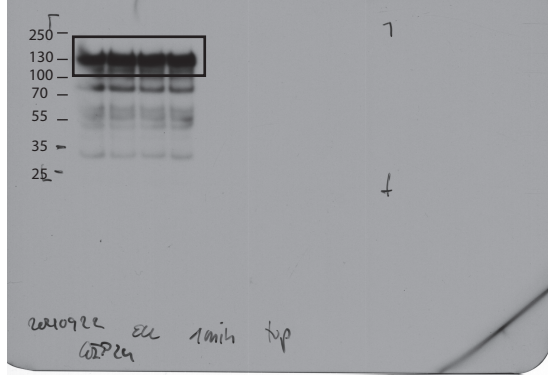

Short exposure

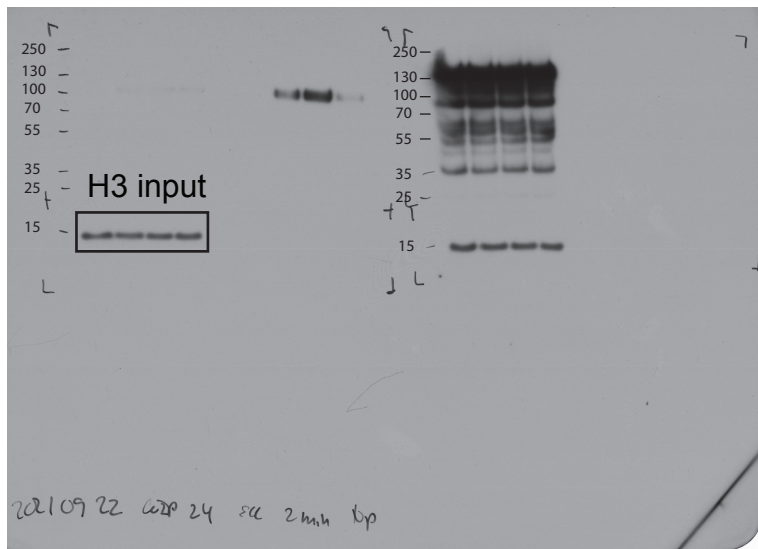

Mid exposure

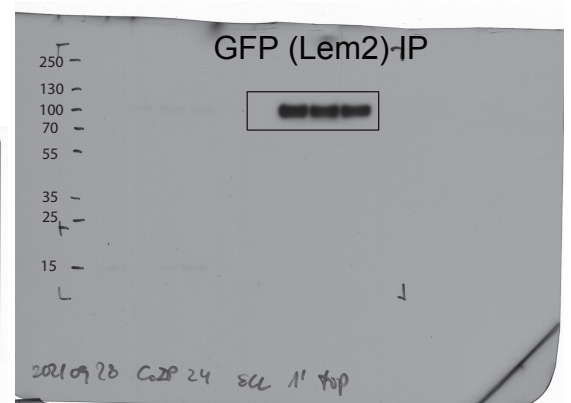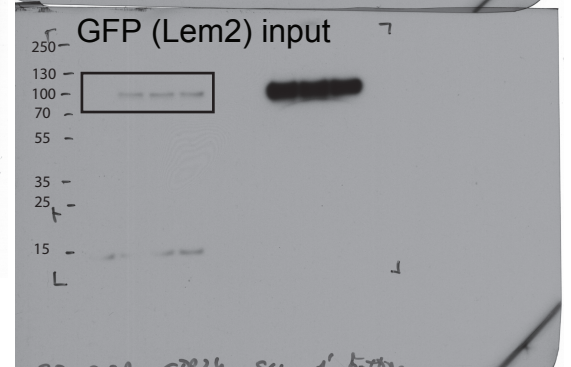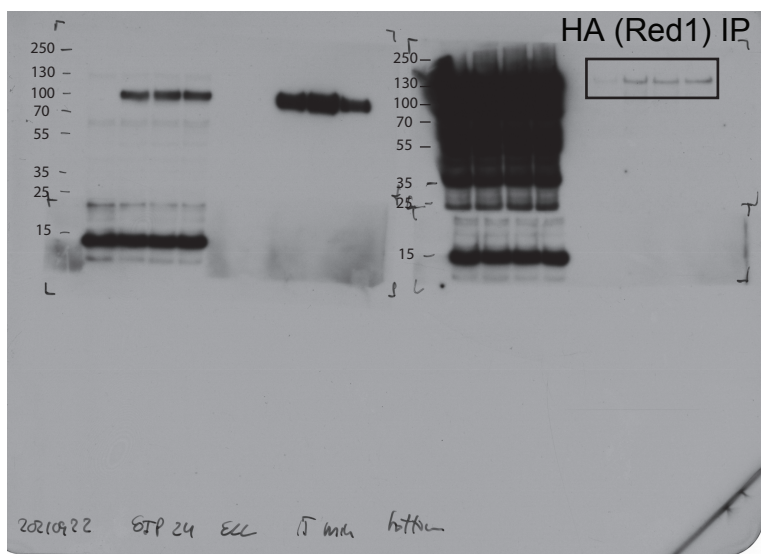

Long exposure

**Fig. 3a**

Short exposure

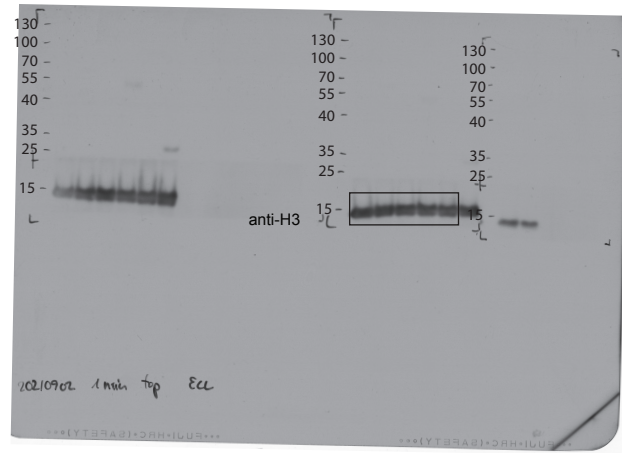

Long exposure

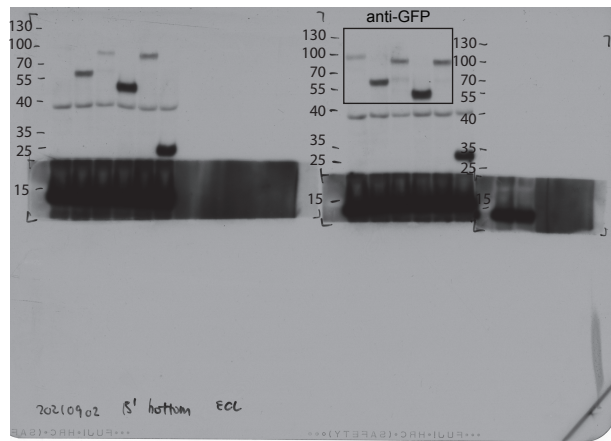

**Fig. 3e**

Supplement: Source Data Fig. 3 — Unprocessed Western Blots [file 41594_2022_831_MOESM10_ESM.pdf]

short exposure

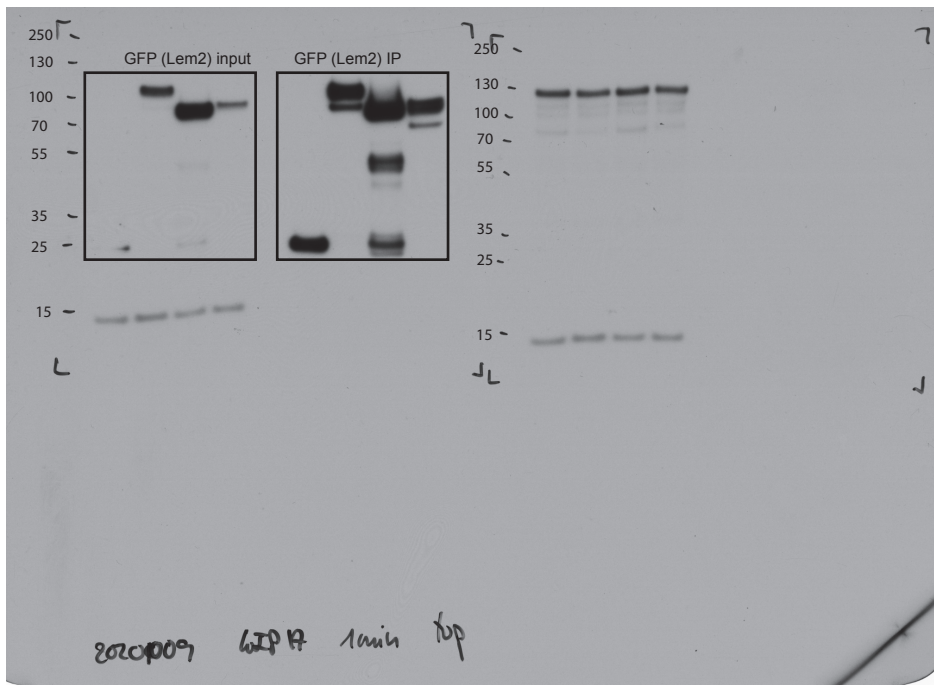

Mid exposure

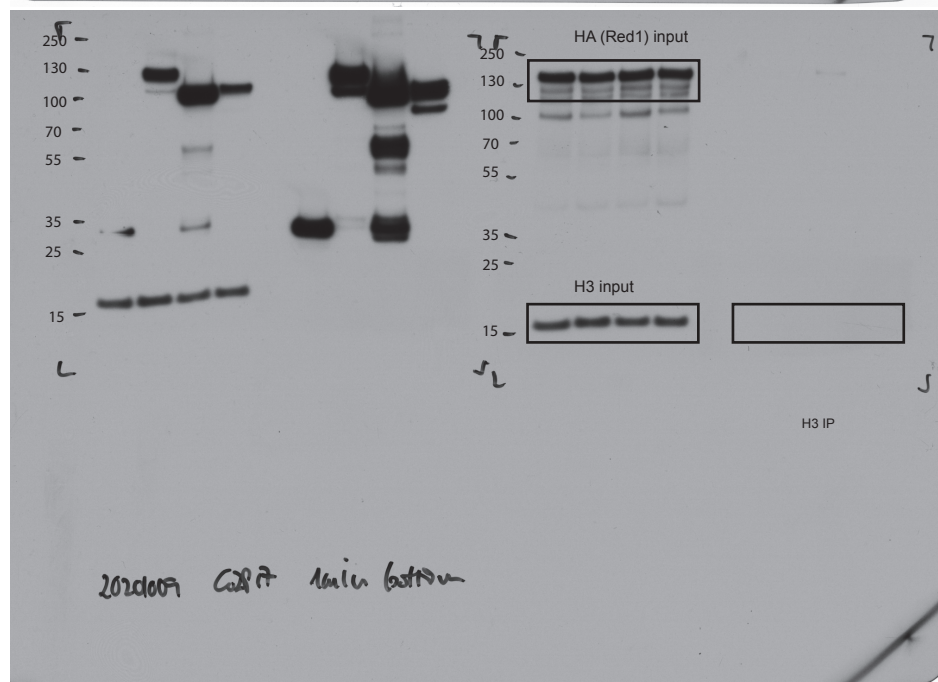

Long exposure

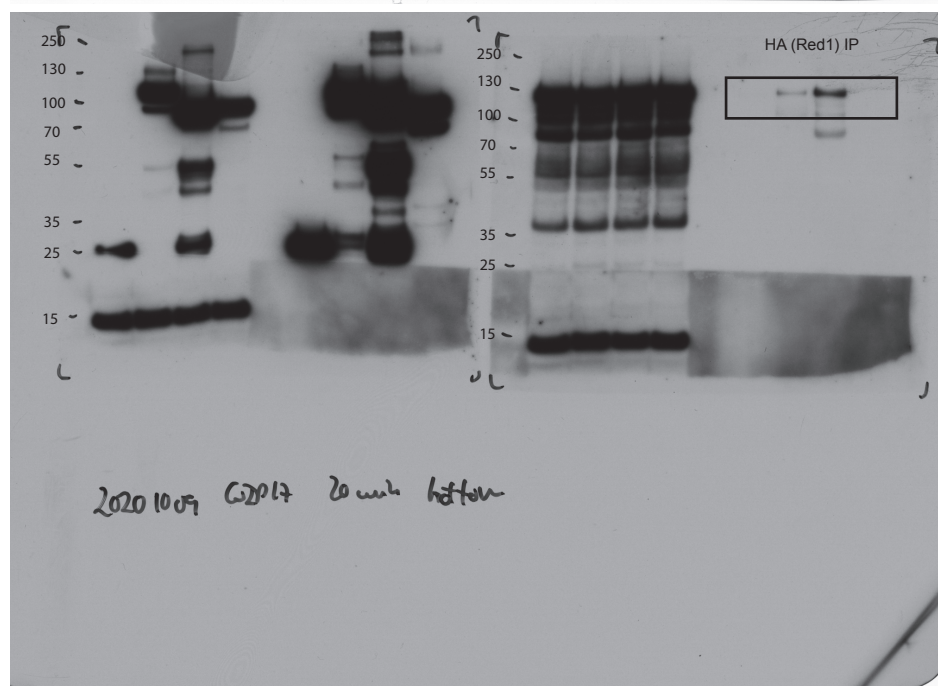

**Extended Data Fig. 3a**

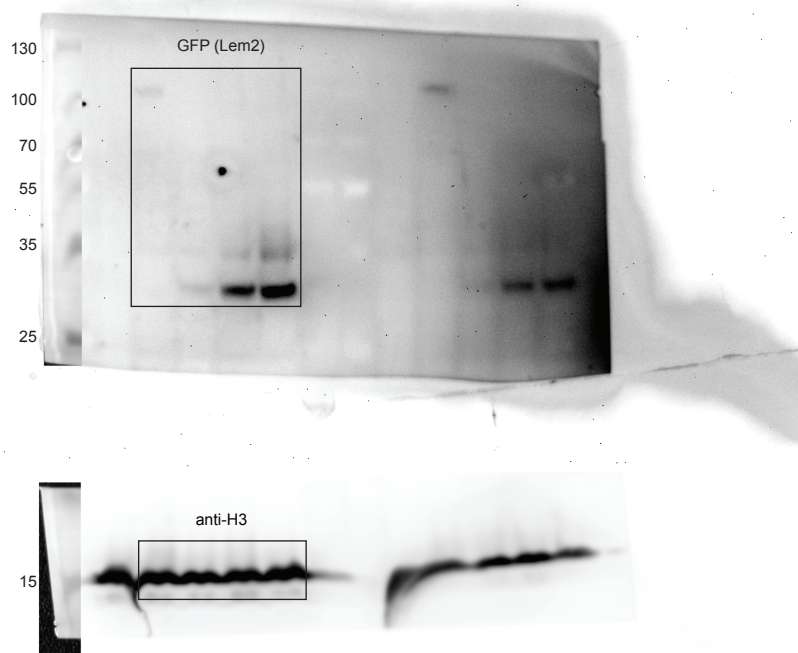

**Extended Data Fig. 3e**

Supplement: Source Data Extended Data Fig. 3 — Unprocessed Western Blots [file 41594_2022_831_MOESM16_ESM.pdf]

Blot 1

Short exposure

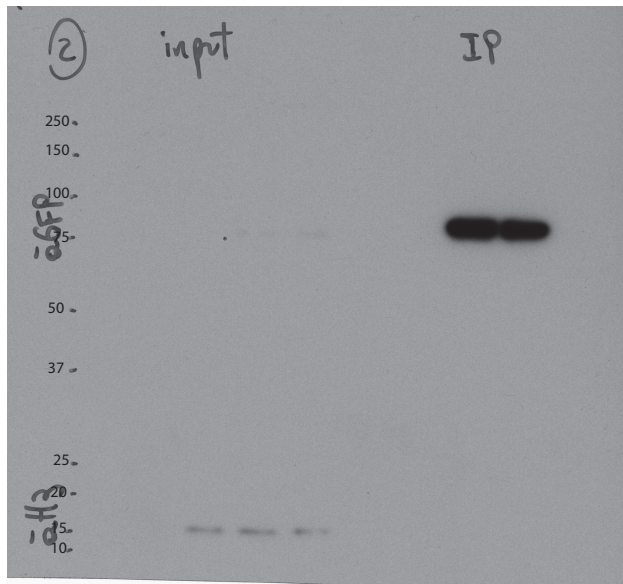

Blot 2

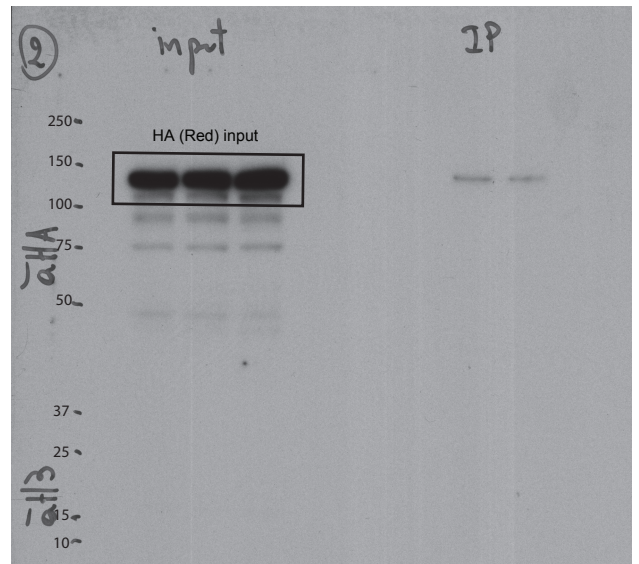

Long exposure

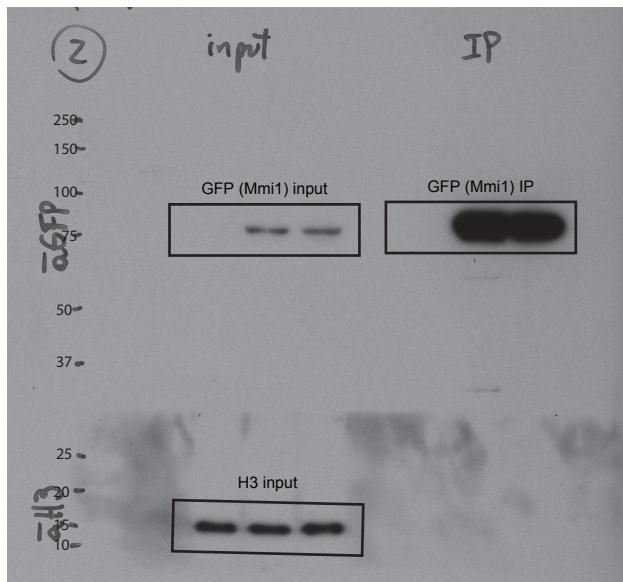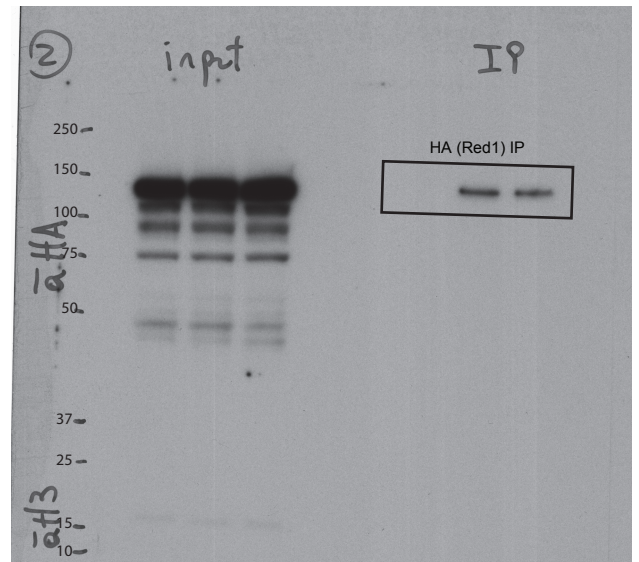

# Extended Data Fig. 7b

Supplement: Source Data Extended Data Fig. 7 — Unprocessed Western Blots [file 41594_2022_831_MOESM21_ESM.pdf]
